# YT521-B homology domain family proteins as N6-methyladenosine readers in tumors

**DOI:** 10.3389/fgene.2022.934223

**Published:** 2022-08-09

**Authors:** Heng Yang, Chengyao Chiang, Qinhong Luo, Chunlan Chen, Junrong Huang, Lizhi Zhu, Duo Zheng

**Affiliations:** ^1^ Guangdong Provincial Key Laboratory of Genome Stability and Disease Prevention, Shenzhen University International Cancer Center, Department of Cell Biology and Genetics, School of Medicine, Department of Pharmacy, The First Affiliated Hospital of Shenzhen University, Shenzhen Second People’s Hospital (Shenzhen Institute of Translational Medicine), Guangdong Key Laboratory for Biomedical Measurements and Ultrasound Imaging, Shenzhen University, Shenzhen, China; ^2^ Central Laboratory, Southern University of Science and Technology, Yantain Hospital, Shenzhen, China

**Keywords:** M6A, YTHDFs, tumor, immune escape, EMT, chemotherapy resistance

## Abstract

N6-methyladenosine (m6A) is the most abundant internal chemical modification of eukaryotic mRNA and plays diverse roles in gene regulation. The m6A modification plays a significant role in numerous cancer types, including kidney, stomach, lung, bladder tumors, and melanoma, through varied mechanisms. As direct m6A readers, the YT521-B homology domain family proteins (YTHDFs) play a key role in tumor transcription, translation, protein synthesis, tumor stemness, epithelial–mesenchymal transition (EMT), immune escape, and chemotherapy resistance. An in-depth understanding of the molecular mechanism of YTHDFs is expected to provide new strategies for tumor treatment. In this review, we provide a systematic description of YTHDF protein structure and its function in tumor progression.

## Introduction

N6-methyladenosine (m6A) is formed by methylation of adenosine at the N6 position. This plentiful posttranscriptional RNA modification extensively regulates multiple aspects of gene expression (He et al., 2019). It exists widely in eukaryotic mRNA and lncRNA and is involved in cell development, stem cell characteristic maintenance, tumor progression, sperm receiving ability, and T cell homeostasis (Dominissini et al., 2012; Maity et al., 2016; Cui et al., 2017; Ianniello et al., 2018). The dynamic m6A RNA modification is catalyzed by the methyltransferase complex (m6A “writer”), demethylases (m6A “eraser”), and m6A-binding proteins (m6A “readers”) (Helm et al., 2017; Meyer et al., 2017). M6A readers, including YT521-B homology (YTH)–containing domain family proteins, insulin-like growth factor binding protein 2, eukaryotic initiation factor, and heterogeneous nuclear ribonucleoproteins (Gilbert et al., 2016), recognize and bind RNA methylation modifications to mediate the translation and degradation of downstream RNA (Wu et al., 2018). Accumulating evidence supports the influence of the m6A modification on lung cancer, melanoma, gastric cancer, bladder cancer, and liver cancer through various mechanisms (Dai et al., 2018; Burbage et al., 2019; Chen X. et al., 2019; Li et al., 2019). In this review, we focus on the current status of our understanding of m6A readers. We describe the functions of YT521-B homologous domain family proteins (YTHDFs) in tumorigenesis and cancer progression. At last, we depict future research directions to fully characterize YTHDFs.

## Biological characteristics of YTHDFs and m6A modification

**FIGURE 1:**
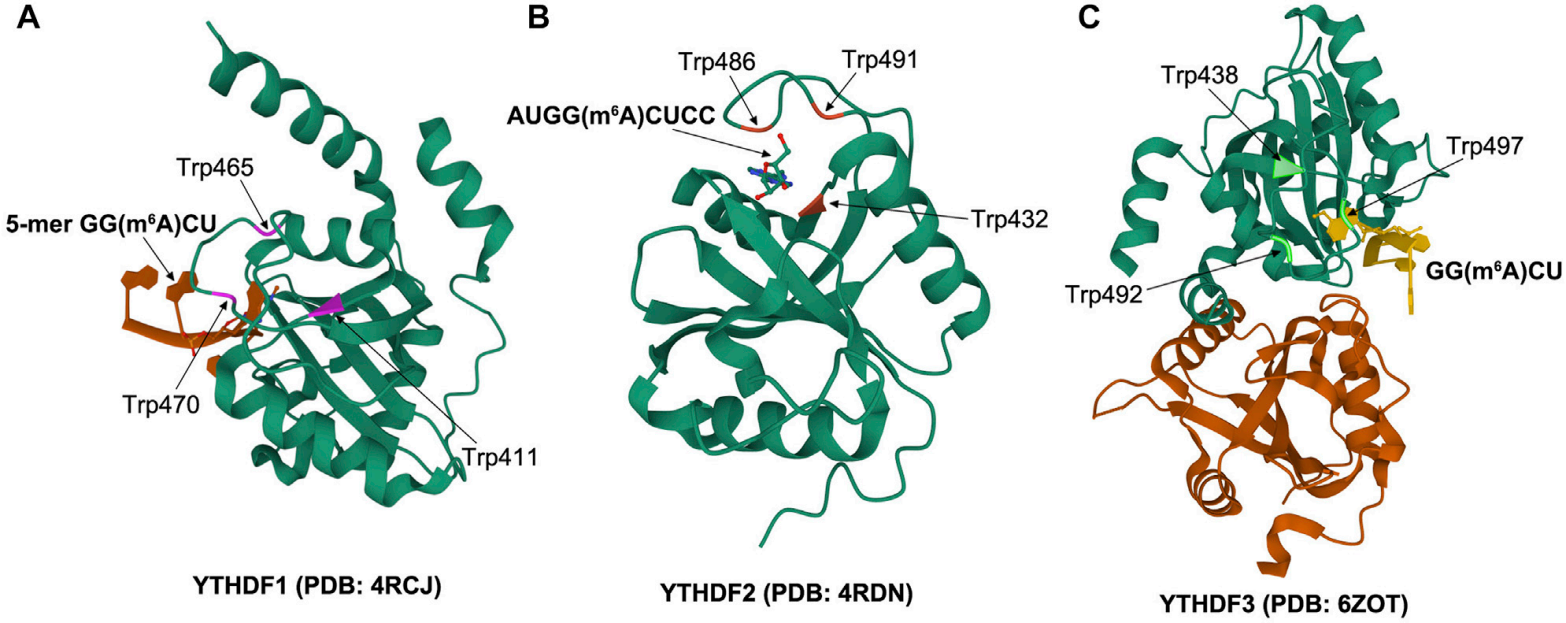
Schematic of crystal structure: **(A)** structure of YTHDF1 YTH domain in complex with 5-mer GG (m6A)CU (PDB id: 4RCJ), **(B)** structure of YTHDF2 YTH domain with AUGG (m6A)CUCC (PDB id: 4RDN), **(C)** structure of YTHDF3 YTH domain in complex with GG (m6A)CU (PDB id: 6ZOT).

As the most common m6A readers, YTH domain–containing proteins include the YTHDF and YTHDC subtypes, which specifically recognize m6A *via* an evolutionarily conserved aromatic cage in the YTH domain (Xu et al., 2014; Shi et al., 2017; Liao et al., 2018). Another two proteins, FMRP translational regulator 1, associated with embryonic development, and leucine-rich pentatricopeptide repeat containing, associated with human tumors, can also recognize m6A modification sites (Arguello et al., 2017; Edupuganti et al., 2017). As the main YTHDF proteins, YTHDF1, YTHDF2, and YTHDF3 were first discovered in the cytoplasm (Wang et al., 2014). YTHDFs contain two domains: a YTH domain at the C-terminal and the unstructured N-terminal of most remaining proteins, comprising a large and low-complexity domain that contains mainly Gln, Asn, and Pro residues (Stoilov et al., 2002). YTHDFs are prone to forming liquid–liquid phase separation and protein droplets because of the presence of this type of large low-complexity domain, especially those with RNA binding domains (Aguzzi et al., 2016; Ries et al., 2019). In fact, proteomic analysis revealed that each YTHDF exists in the form of droplet structures, involving stress particles and artificially created RNA particles (Kato et al., 2012; Jain et al., 2016). The m6A binding pocket of the YTHDF1 YTH domain is made up of two α helices (α1 and α2) and four β strands (β1, β2, β4, and β5). To be exact, m6A is properly matched in a pocket composed of Trp411, Trp465, and Trp470, parallel to the loop planes of Trp411 and Trp470 and perpendicular to the loop plane of Trp465 (Xu et al., 2015) (Figure 1A). The residues Lys416 and Arg527 of the YTHDF2 YTH domain play a critical role in binding RNA backbone and hydrophobic vesicles. M6A single nucleotide is located in the aromatic cage composed of three aromatic amino acids Trp432, Trp486, and Trp491, forming π–π interaction. These types of particular binding contribute to the specific recognition of m6A (Ma et al., 2019) (Figure 1B). YTHDF3 possesses 86% sequence identity with the YTH structural domain of both YTHDF1 and YTHDF2. Furthermore, X-ray structural analyses and molecular dynamics simulations demonstrated that all YTHDF YTH domains share essentially equal interactions with the m6A-modified RNA and have an analogous affinity (Figure 1C), suggesting the redundant characters of the three proteins in physiological functions (Li et al., 2020). Different binding proteins selectively recognize m6A-modified RNA and exert a crucial role in regulating target gene expression. YTHDF1 increases the transmission of mRNA transcription complex by binding to m6A at a site around the stop codon and coordinates translation initiation and protein synthesis (Wang et al., 2015). YTHDF2 was the first binding protein recognized to regulate mRNA stability and remodeling of RNA structure through m6A modification (Ivanova et al., 2017). YTHDF3 is usually coupled with YTHDF1 and YTHDF2, which control the attenuation or translation of methylated RNA (Li et al., 2017). Although many studies have provided evidence of the importance of the m6A recognition protein in multiple physiological and pathological processes, further studies are still needed to fully clarify its role in mRNA biology.

## YTHDFs and tumorigenesis

YTHDFs have been reported to elevate the growth and metastasis of various tumors, such as hepatic cell carcinoma (HCC), gastric cancer (GC), colorectal cancer (CRC), nonsmall cell lung cancer (NSCLC), and triple-negative breast cancer (TNBC) (Table 1). For example, YTHDF1 expression was dramatically upregulated in stage III and IV HCC patients compared with stage II disease, and the prognosis of patients with high YTHDF1 levels was worse (Zhao et al., 2018). Since the potential target genes regulated by YTHDF1 might be associated with amino acid degradation and lipid metabolism in the tumor cell cycle, abnormalities in these physiological functions could lead directly to the occurrence and progression of HCC. YTHDF2 also acts as an oncogene to promote the HCC lung metastasis and cancer stem cell (CSC) phenotype through increasing m6A methylation levels of OCT4 mRNA in the 5′-untranslated region (UTR) and OCT4 protein expression (Zhang et al., 2020). In addition, YTHDF2 attaches to m6A sites to boost mRNA degradation. When the m6A modification level in tumor suppressor gene SOCS2 mRNA was upregulated, the increased number of m6A binding sites in YTHDF2 ultimately promoted the degradation of SOCS2 mRNA, which was beneficial to HCC cell proliferation (Chen et al., 2018). These studies reveal that YTHDFs can accelerate HCC progression by promoting protein translation and regulating the stability of mRNA.

**TABLE 1:** YT521-B homology domain family proteins in tumorigenesis.

Reader	Trend	Tumor	Regulatory pathway/Gene	References
YTHDF1	↑	HCC	Cell cycles	Zhao et al. (2018)
	↑	GC	Wnt/β-catenin, FZD7	Pi et al. (2021)
	↑	CRC	RhoA Signaling, ARHGEF2	Wang et al. (2022)
	↑	NSCLC	CDK2, CDK4, cyclin D1	Shi et al. (2019)
		TNBC	ITGA6	Jin D. et al. (2019)
	↑	GBM		Xu et al. (2020)
YTHDF2	↑	HCC	OCT4, SOCS2	Zhang et al. (2020); Chen et al. (2018)
	↑	GC	Cell cycle, apoptosis	Zhang et al. (2017)
	↑	NSCLC	6-PGD	Sheng et al. (2020)
	↑	BCa	SETD7, KLF4	Zheng et al. (2021); Xie et al. (2020)
	↑	GBM	LXRα, HIVEP2	Fang et al. (2021)
YTHDF3	↑	CRC	GAS5, YAP	Ni et al. (2019)
	↑	TNBC	ZEB1, EMT	Lin et al. (2022)

GBM, glioma; BCa, bladder cancer.

Huo et al. (2021) reported that YTHDF1 promoted upregulation of SPHK2 expression at the protein level *via* its specific structural domain, whereas SPHK2 coupled with KLF2 to induce phosphorylation modifications, thereby mediating KLF2 degradation and promoting the antiapoptotic function of GC cells. Compared with normal tissues, YTHDF2 was significantly upregulated in gastric cancer, Silencing of YTHDF2 suppressed gastric cancer cell multiplication, arresting the cell cycle in the G1/S phase and boosting cell apoptosis (Zhang et al., 2018).

In CRC, c-MYC initiated YTHDF1 expression, and high expression was associated with poor overall survival (OS) (Nishizawa et al., 2018). LncRNA-GAS5 was shown to directly bind to the WW domain of YAP to promote its phosphorylation and subsequently ubiquitin degradation, thus declining YTHDF3 transcription. YTHDF3 tended to selectively combine with GAS5 in methylated m6A site, thereby initiating its decay and the formation of a GAS5-YAP-YTHDF3 negative feedback loop to accelerate CRC development (Ni et al., 2019). This study suggests that targeting the lncRNA GAS5-YAP-YTHDF3 axis is a promising approach for CRC treatment.

In a large-scale genome and transcriptome sequencing, Shi et al. (2019) found that YTHDF1 was amplified in NSCLC from Tibetan domestic mammals. Knockdown of YTHDF1 inhibited NSCLC cell growth and the formation of xenograft tumor by adjusting the translational efficiency of CDK2, CDK4, and cyclin D1 and also restrained *de novo* lung adenocarcinoma progression. It is of interest that patients with high expression of YTHDF1 in high altitudes have a better clinical prognosis. This is because the knockdown of YTHDF1 activates the Keap1-Nrf2-AKR1C1 axis, which increases the sensitivity of NSCLC patients to cisplatin chemotherapy. The above results indicate that the specific recognition and regulation of YTHDF1 on m6A-modified mRNA may be associated with time and cell environment, which contributes to different functions of YTHDF1 in various tumors. Moreover, YTHDF2 was reported to be upregulated in lung cancer and shown to bind straightly to the m6A modification site in the 3′-UTR of the 6-phosphogluconate dehydrogenase (6-PGD) mRNA. This event promoted the translation of 6-PGD mRNA and enhanced the pentose phosphate pathway flux to facilitate the growth of lung cancer cells (Sheng et al., 2020).

It was found that m6A abundantly existed in ITGA6 transcripts, and methyltransferase-like 3 (METTL3) promoted YTHDF1/YTHDF3 binding to the ITGA6 mRNA 3′-UTR, thereby enhancing ITGA6 mRNA translation and changing the adhesion ability of bladder cancer cells (Laudato et al., 2017; Jin H. et al., 2019). In TNBC patients, YTHDF3 expression was correlated with poorer disease-free survival (DFS) and OS. YTHDF3 improved ZEB1 mRNA stability in an m6A-dependent manner and positively regulated cell migration, invasion, and EMT in TNBC cells (Lin et al., 2022). The above evidence demonstrates that YTHDF3 can not only function alone but also couple with METTL3 to elevate the translation of methylated RNA, playing a vital role in tumor progression.

It is worth noting that some studies have shown that YTHDF1 inhibits tumor progression. Knockdown of YTHDF1 could improve the crosspresentation ability of dendritic cells in mouse melanoma, and when combined with a PD-L1 checkpoint inhibitor, the tumor was almost completely controlled (Han et al., 2019). Another study showed that m6A modification dramatically inhibited ocular melanoma cell growth, and its low expression suggested a poor prognosis. YTHDF1 functions as a tumor suppressor of ocular melanoma by recognizing m6A-modified RNA and accelerating the translation of histidine trinucleotide binding protein 2 (HINT2) (Jia et al., 2019). Hence, as m6A readers, YTHDFs regulate tumor-specific oncogene expression and biological function *via* different mechanisms. Thus, targeting YTHDFs is implicated as a new strategy for cancer treatment.

## YTHDFs and tumor immune responses

T cell immune responses play important roles in tumor immunotherapy. Although neoantigens in cancer patients are abundant, thorough removal of tumors is hindered by the failure to produce lasting antitumor immune responses (Yarchoan et al., 2017). Han et al. (2019) reported that YTHDF1 regulated neoantigen-specific immunity *via* m6A modification. YTHDF1 depletion in classical dendritic cells not only increased the mutual expression of tumor antigens but also enhanced CD8^+^ T cells crosspriming. The transcripts of lysosomal proteases were labeled by m6A and recognized by YTHDF1, thus promoting the translation of lysosomal cathepsin in dendritic cells and significantly inhibiting crosspresentation by wild-type dendritic cells. This study also showed that T cells in YTHDF1-deficient mice produced high levels of IFN-γ, indicating that YTHDF1 knockout in host cells promotes T cell activation at an early stage. In addition, the therapeutic effect of a PD-L1 immune checkpoint inhibitor was heightened in YTHDF1^−/−^ mice, with almost complete inhibition of tumor growth, suggesting the potential importance of YTHDF1 as a therapeutic target for antitumor immune therapy.

Lin et al. (2020) found that YTHDF2 expression was positively associated with the expression of multiple immune checkpoint biomarkers, including CTLA-4, TIM-3, and PD-1, as well as isocitrate dehydrogenase 1, and tumor-associated macrophage factors in lower-grade glioma. Further experiments are needed to confirm this bioinformatic conclusion. In addition, high YTHDF2 transcription was associated with a higher survival rate and increased tumor-infiltrating lymphocytes in clear cell renal cell carcinoma and NSCLC patients, suggesting the antitumor function of YTHDF2 (Su et al., 2021; Tsuchiya et al., 2021). It is of interest that there are two aspects to the regulatory role of YTHDF2 in tumor immunity. Wang et al. (2020) reported that YTHDF2 stabilized STAT1 and IRF1 mRNA in tumors deficient in METTL3 or METTL4, thus promoting the signal transduction of the IFN-γ-STAT1-IRF1 axis and enhancing the response of immunotherapy-resistant colorectal cancer to PD-1 treatment. Methylated YTHDF2 decreased the stability of mRNA transcribed from the melanoma-promoting genes PD-1, CXCR4, and SOX10, to inhibit the growth of melanoma (Yang et al., 2019). A natural killer (NK) cell is a type of lymphocyte that mediates antitumor immune response. Ma et al. (2021) showed that YTHDF2 was upregulated in NK cells to maintain the steady state and terminal maturation of NK cells after activation of the immune system. In a mechanistic manner, YTHDF2 mediated the release of IFN-γ, granzyme B, and perforin in NK cells by forming a STAT5-YTHDF2 positive feedback loop, which finally promoted TARDBP degradation and enhanced NK proliferation and survival. However, YTHDF2 was found to inhibit innate immunity by binding with m6A-modified circZKSCAN1 in mammalian cells, to inhibit RIG-I recognition and k63-PolyUb and promote tumor immune escape (Chen Y. G. et al., 2019). Therefore, YTHDF2 not only enhances immunity but also promotes tumor immune escape, which are functions that may depend on differences in tumor types and the mechanisms of tumorigenesis.

Furthermore, some studies have shown that YTHDF3 contributes to the formation of the tumor microenvironment and could be used as an immune-related marker. High YTHDF3 expression indicates poor survival with a diversity of lymphocyte infiltration in breast cancer, as well as esophageal squamous cell carcinoma and head and neck squamous cell carcinoma (Zhang et al., 2021; Zhao et al., 2021; Pu et al., 2022). The above research shows that YTHDF3 may be a potential therapeutic target related to tumor-infiltrating immune cells.

## YTHDFs and EMT

EMT is the key process of tumor cell metastasis, with the deficit of E-cadherin, which is considered to be the most basic event in this process. As a key transcription factor in this process, Snail reduces the expression of E-cadherin, which promotes tumor recurrence, metastasis, and drug resistance (Jang et al., 2019). YTHDF1 promotes cancer progression by regulating EMT. Lin et al. (2019) found that Snail was methylated in the CDS region and that YTHDF1 was significantly enriched in the CDS region of Snail mRNA leading to increased TGF-β-induced snail expression in HeLa cells. In addition, YTHDF1 silencing significantly increased the expression levels of Claudin-1 and zonula occludens protein 1, whereas expression of matrix metalloproteinase-9, matrix metalloproteinase-2, Vimentin, and N-cadherin was inhibited. These findings indicated that YTHDF1-mediated EMT is responsible for promoting the migration and invasion of HCC cells (Luo et al., 2021).

Recent studies have confirmed the involvement of YTHDF2 in tumor EMT and its role in regulating tumor cell migration and invasion. The METTL3/YTHDF2 m6A axis regulates miR-1915-3p expression by inhibiting the activity of the transcription factor KLF4, thereby targeting SET and inhibiting NSCLC cell migration, invasion, and EMT (Pan et al., 2021). Chen et al. (2017b) reported that YTHDF2 orchestrated two cellular processes in pancreatic cancer cells. YTHDF2 promoted pancreatic cancer cell proliferation and inhibited migration and invasion, a phenomenon termed “migration-proliferation dichotomy,” and also repressed EMT, probably by negatively regulating the YAP signaling pathway (Supplementary Figure S1). Therefore, YTHDF2 should be seriously considered as a therapeutic target for pancreatic cancer. Other studies showed that YTHDF3 promotes tumor migration and invasion by regulating EMT. Knockdown of YTHDF3 increased E-cadherin expression and reduced the expression of N-cadherin and Vimentin, which finally inhibited the migration and invasion of TNBC cells (Lin et al., 2022). Wang M. et al. (2020) reported that YTHDF3 improved ZEB1 mRNA stability in an m6A-dependent manner, maintained ZEB1 mRNA stability, and finally, promoted the translation and expression of ZEB1.

## YTHDFs and drug resistance in tumors

Abnormal AKR1C1 expression induces the development of drug resistance in chemotherapy of cancers including NSCLC, breast cancer, and ovarian cancer (Chen et al., 2017a). YTHDF1 knockout was associated with decreased Keap1 expression, whereas the expression of Nrf2 and its downstream factor AKR1C1 was increased. Furthermore, YTHDF1 knockout reduced the translation efficiency of m6A-modified Keap1 transcripts, resulting in activation of the antioxidant oxygen radical scavenging system (Nrf2-AKR1C1) and resistance of NSCLC to cisplatin chemotherapy, leading to a worse clinical prognosis. This study suggested that the Keap1-Nrf2-AKR1C1 axis was the downstream regulatory target of YTHDF1, emphasizing the important character of YTHDF1 in hypoxia adjustment and NSCLC progression (Shi et al., 2019). In addition, YTHDF1 was recruited to the m6A modification site of TRIM29, promoting TRIM29 translation in cisplatin-resistant ovarian cancer cells. Furthermore, YTHDF1 knockdown inhibited cisplatin-resistant ovarian cancer cells with CSC-like characteristics (Hao et al., 2021), suggesting that YTHDF1 is a promising cancer marker to trace the recurrence of ovarian cancer.

Studies have shown that temozolomide (TMZ) resistance was acquired in recurrent glioblastoma multiforme (GBM) tumors after radiotherapy and chemotherapy. After 60 Gy radiation, YTHDF2 synthesis in GBM cells was significantly upregulated (Pak et al., 2021), suggesting that YTHDF2 plays an important role in TMZ resistance to recurrent glioblastoma. Huang et al. (2019) showed that TUSC7, a strong inhibitory lnc-RNA, was expressed at low levels in NSCLC tissues and inhibited the nondivision stem cells by obstructing Notch signaling. YTHDF2 promoted the renewal of the stem cell–like population in erlotinib-resistant cells by inhibiting TUSC7 and promoted the characteristics of tumor resistance (Li et al., 2021). Furthermore, YTHDF1/3 increased the drug resistance of tumors by promoting the translation of drug resistance–related proteins. Jin et al. (2019) found that METTL3 improved the m6A modification levels of YAP and further accelerated YAP translation by engaging YTHDF1/3 and the eEIF3a translation initiation complex, thereby inducing the NSCLC drug resistance and metastasis. Therefore, there is strong evidence that YTHDFs play an important regulatory role in the responses of tumors to chemotherapy and represent a promising therapeutic target for drug-resistant cancer.

## Conclusion and perspective

Based on the current research, YTHDFs mainly recognize m6A-modified target genes, increase translation through triggering translational initiation and elongation, and then control mRNA stability and target gene expression. YTHDFs affect tumorigenesis, metastasis, tumor immunity, EMT, and chemoresistance by regulating target gene expression. Many studies have shown the various functions of YTHDFs and further revealed the crosstalk and competition between different YTHDFs. Here, we summarized the current status of the structure and biological characteristics of m6A readers—YTHDFs—in human cancers. However, a full and comprehensive understanding of YTHDFs remains a distant prospect for several reasons. First, YTHDFs exert either oncogenic or tumor-suppressive effects that may depend on the type of regulated targets or interactome in specific cancer species. Second, the biological significance of liquid–liquid separation induced by the low-complexity domain of YTHDFs in tumors is still unknown. Third, we need to dissect the interactions among YTHDFs as well as those between YTHDFs and erasers and readers in tumorigenesis, metastasis, and immunity of human cancers. Fourth, we need to determine how the expression and activity of YTHDFs are regulated in cancer and whether the recognition of m6A by YTHDFs is RNA sequence specific. At last, it is unclear whether YTHDFs can regulate the occurrence and development of cancer by recognizing m6A-modified noncoding RNA, such as microRNA or lncRNA. The functions of YTHDF mutants and isoforms in tumor molecular diagnosis also represent an important topic for future research.

Several groups have reported that targeting m6A-modifying enzymes and regulatory proteins, such as FTO (Su et al., 2020), METTL3 (Yankova et al., 2021), and ALKBH5 (Fang et al., 2022), is beneficial to the treatment of malignant leukemia and solid tumors. We also found a covalent small molecule drug, which could activate the tumor immune microenvironment and play an antihepatoma role by targeting the m6A recognition protein IGF2BP1 (Liu et al., 2022). Thus, the targeted regulation of YTHDFs by small chemical molecules may be a promising therapeutic strategy for human cancer. Overall, more basic and clinical studies are required to fully elucidate the mechanism underlying the functions of YTHDFs, which will provide new hope for cancer diagnosis and targeted treatment.
