# Supplementary figures and images for: YT521-B homology domain family proteins as N6-methyladenosine readers in tumors
Source: Front Genet. 2022 Aug 9;13:934223. doi: 10.3389/fgene.2022.934223 (PMC9395638; doi:10.3389/fgene.2022.934223)

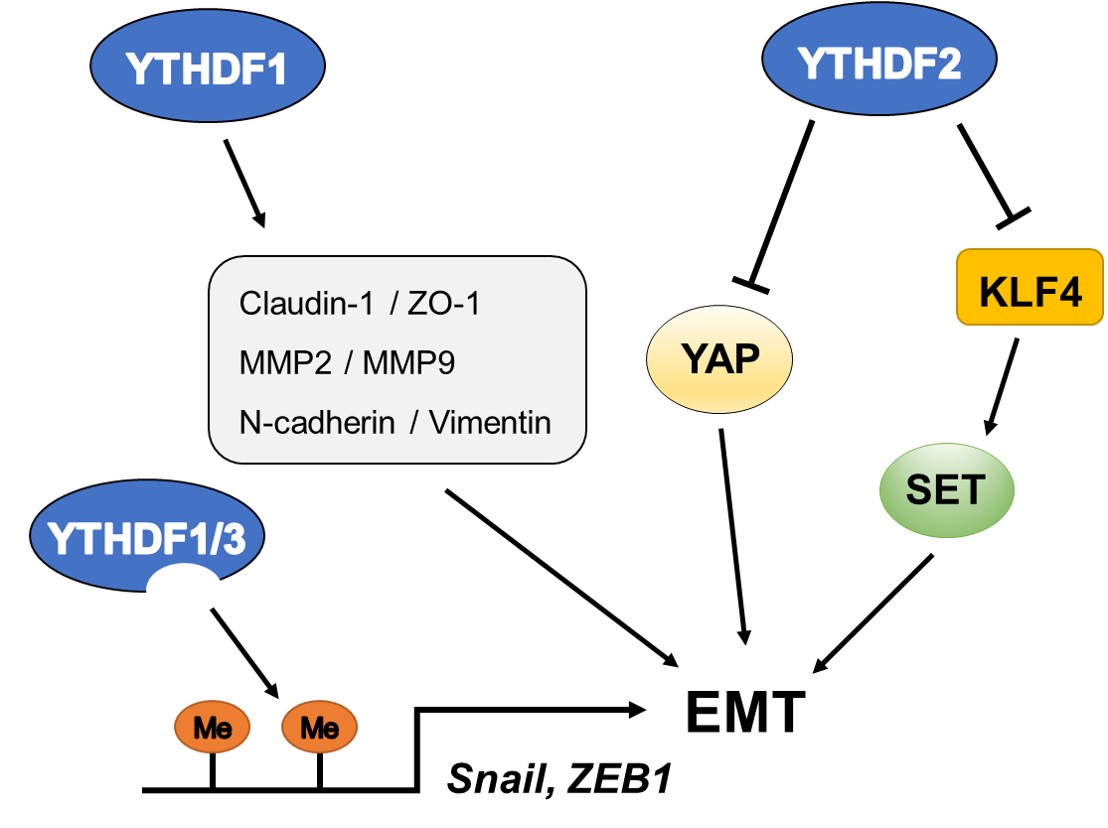

Supplement: Supplementary file 1 [file Image1.JPEG]
